# A proteome-wide systems toxicological approach deciphers the interaction network of chemotherapeutic drugs in the cardiovascular milieu†

**DOI:** 10.1039/c8ra02877j

**Published:** 2018-06-04

**Authors:** Suvendu Giri, Jeganathan Manivannan, Bhuvaneswari Srinivasan, Lakshmikirupa Sundaresan, Palanivel Gajalakshmi, Suvro Chatterjee

**Affiliations:** Department of Biotechnology, Anna University Chennai Tamil Nadu India soovro@yahoo.ca; Vascular Biology Lab, AU-KBC Research Centre, MIT Campus of Anna University Chennai Tamil Nadu India; Environmental Health and Toxicology Lab, Department of Environmental Sciences, Bharathiar University Coimbatore Tamil Nadu India

## Abstract

Onco-cardiology is critical for the management of cancer therapeutics since many of the anti-cancer agents are associated with cardiotoxicity. Therefore, the major aim of the current study is to employ a novel *in silico* method combined with experimental validation to explore off-targets and prioritize the enriched molecular pathways related to the specific cardiovascular events other than their intended targets by deriving relationship between drug-target-pathways and cardiovascular complications in order to help onco-cardiologists for the management of strategies to minimize cardiotoxicity. A systems biological understanding of the multi-target effects of a drug requires prior knowledge of proteome-wide binding profiles. In order to achieve the above, we have utilized PharmMapper, a web-based tool that uses a reverse pharmacophore mapping approach (spatial arrangement of features essential for a molecule to interact with a specific target receptor), along with KEGG for exploring the pathway relationship. In the validation part of the study, predicted protein targets and signalling pathways were strengthened with existing datasets of DrugBank and antibody arrays specific to vascular endothelial growth factor (VEGF) signalling in the case of 5-fluorouracil as direct experimental evidence. The current systems toxicological method illustrates the potential of the above big-data in supporting the knowledge of onco-cardiological indications which may lead to the generation of a decision making catalogue in future therapeutic prescription.

## Abstract 2

Onco-cardiology is critical for the management of cancer therapeutics since many of the anti-cancer agents are associated with cardiotoxicity.

## Graphical abstract

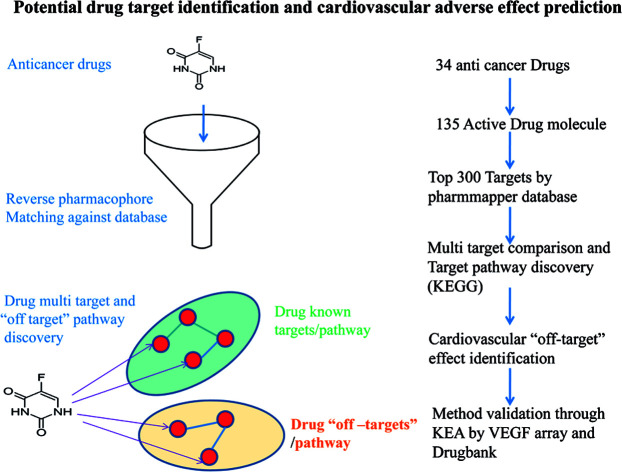

## Introduction

Chemotherapeutic agents used in the treatment of cancer are often associated with many off-target effects among which the cardiovascular system is highly affected, accounting to the increased mortality of patients.^1^ The cardiotoxicity associated with anti-cancer therapy can be grouped into several major categories including myocardial dysfunction, congestive heart failure (CHF), coronary artery disease, cardiac systolic dysfunction, arrhythmias, cardiac ischemia, pericarditis and chemotherapy induced repolarization abnormalities in addition to the direct cytotoxic effects of the drugs.^2^ There has been a 3.5-fold increased mortality risk of CHF induced by chemotherapy as compared to idiopathic cardiomyopathy.^3^ There have been predictions that the risk of death from cardiovascular diseases (CVD) may exceed the risk of recurrence of cancer.^4,5^

It is not only the primary drug molecule that acts on the biological system but also the derivatives that originate as by-products of different enzymatic reactions (oxidation, reduction, hydroxylation, deglycosidation, deamination, demethylation, dealkylation, *etc.*) in the biological system that cause cardiotoxicity.^6^ For example, the metabolites of doxorubicin and 5-fluorouracil (5-FU), namely doxorubicinol and fluorodeoxyuridine monophosphate (FdUMP) respectively, are more cardiotoxic than their respective parent molecules.^7,8^ Therefore, it is essential to study the effects of metabolites along with the parent molecules.

The cardiotoxic effects of anti-cancer drugs seem to be majorly due to their promiscuous effects on the physiological system. In order to understand the basis of side-effects of the drugs, we need to explore and predict the so-called off-target or side effects. The need for prediction and management of cardiovascular effects of drugs is compelling. As individual target screening of anti-cancer drugs through biological experiments would be a haunting task, *in silico* approaches may provide solutions to a great extent.^9,10^ Plentiful computational methods have been developed for predicting the interaction between enzymes and drugs in the cellular networks. Notably iEzy-Drug,^11^ iGPCR-Drug.^12^ iCDI-PseFpt,^13^ iNR-Drug,^14^ iDrug-Target^15^ predict the protein–drug interaction and its corresponding network as discussed.^16^ Among the many distinct algorithms, we have chosen the PharmMapper – a web server that identifies potential drug targets *via* large-scale reverse pharmacophore mapping strategy.^17^ With the use of PharmMapper, a robust and efficient mapping method, we attempted to predict the targets of selected 135 active drug components including thirty four mother drug molecules. With the enriched targets, as predicted by the PharmMapper, we investigated the compound–targets–pathway relationship which would be of aid in predicting the side-effects of a given drug.^18^ In addition, we wanted to explore the basis behind the fact that despite having different molecular mechanisms, most of the anti-cancer drugs cause toxic effects in the cardiovascular system. Our *in silico* predictions were validated through existing experimental results (DrugBank) and with phosphorylation array for a selected drug.

## Materials and methods

Human umbilical vein endothelial cells (HUVEC) were obtained from Lonza Group (Headquarters: Basel, Switzerland). VEGF Pathway Phospho Antibody Array was purchased from Full Moon BioSystems, Sunnyvale, USA. 5-Fluorouracil was commercially obtained from Sigma-Aldrich. All other reagents were commercially acquired in analytical reagent grade.

### Identifying CVD drugs & their metabolites

Chemotherapeutic drugs (including its major metabolites) that are believed to be associated with cardio toxicity were identified and their relevant information were obtained through searching previous literature found in PubMed literature. The list of drugs and its metabolites used in the current study are illustrated in Table 1.

**Table 1:** The extensive list of all the study related drug and their metabolites along with their PubChem ID are illustrated

S. no.	Drug name	Active drug component	PubChem/DrugBank ID
1	5-Fluorouracil	5-Fluorouracil	3385
		5-Fluorouridine monophosphate (FUMP)	150856
		5-Fluorouridine diphosphate (FUDP)	46936877
		5-Fluorouridine triphosphate (FUTP)	10255482
		Fluorodeoxyuridine	5790
		5-Fluorodeoxyuridine monophosphate (FdUMP)	46936787
		5-Fluorodeoxyuridine diphosphate (FdUDP)	53882537
		5-Fluorodeoxyuridine triphosphate (FdUTP)	503023
2	Bleomycin	Bleomycin	5360373
		Ferric bleomycin	124117
		Deamidobleomycin	5488286
3	Busulphan	Busulphan	2478
		Methane sulfonic acid	6395
		3-Hydroxysulfolane	98932
4	Camptothecin	Camptothecin	2538
		9-Methoxycamptothecin	123617
		10-Hydroxycamptothecin	97226
5	Carboplatin	Carboplatin	498142
6	Cisplatin	*cis*-Diamminemonoaquamonochloroplatinum ii	171305
		*cis*-Diamminedichloroplatinum-(ii)	2767
7	Cyclophosphamide	Cyclophosphamide	2907
		Dechloroethyl-cyclophosphamide	114861
		4-Hydroxy-cyclophosphamide	99735
		4-Ketocyclophosphamide	33676
		Aldophosphamide	107744
		Phosphoramide mustard	96356
		Carboxy cyclophosphamide	31515
		Iminocyclophosphamide	134773
		4-Glutathionyl cyclophosphamide	443288
8	Cytarabine	Cytarabine	6253
		Cytarabine 5′-triphosphate	25774
		1-(Beta)-d-arabinofuranosyluracil	46780471
9	Dasatinib	Dasatinib	3062316
		Hydroxy methyl Dasatinib	11854534
		*N*-Deshydroxy ethyl Dasatinib	11669430
		Dasatinib N-oxide	11854535
		Dasatinib carboxylic acid	11854012
		Dasatinib alpha d glucuronide	71315192
		Dasatinib beta d glucuronide	71434186
10	Daunorubicin	Daunorubicin	30323
		Daunorubicinol	71668325
		Daunorubicine aglycone (Daunomycinone)	83843
		Daunorubicinol aglycone (Daunomycinolone)	147191
		7-Deoxydaunorubicinone	12831689
		7-Deoxydaunorubicinol aglycone	14563991
11	Docetaxel	Docetaxel	148124
		Docetaxolum	64780
		Hydroxyoxazolidinone	91800159
		Oxazolidinedione	15765782
12	Doxorubicin	Doxorubicin	31703
		Doxorubicinol	83970
		Doxorubicin deoxyaglycone	83958
		Doxorubicin hydroxyaglycone	http://www.drugbank.ca/metabolites/DBMET01078
		Doxorubicinol hydroxyaglycone	http://www.drugbank.ca/metabolites/DBMET01079
		Doxorubicin semiquinone	http://www.drugbank.ca/metabolites/DBMET00846
13	Epirubicin	Epirubicin	41867
		Epirubicinol	127118
		Epirubicin glucuronide	101612255
14	Erlotinib	Erlotinib	176870
		Erlotinib acetic acid	76969213
		*O*-Desmethyl Erlotinib	16045730
		Hydroxy Erlotinib	16045656
		Desmethyl Erlotinib carboxylate acid	71315775
15	Etoposide	Etoposide	36462
		Etoposide catechol	127462
		Etoposide-*ortho*-quinone	71316630
		Etoposide glucuronide	46173784
16	Everolimus	Everolimus	6442177
		Seco Everolimus	71748854
17	Gemcitabine	Gemcitabine	60750
		2′,2′-Difluorodeoxycytidine 5′-triphosphate (DFdCTP)	130659
		2′,2′-Difluoro-2′-deoxycytidine 5′-diphosphate (DFdCDP)	6420157
		2′,2′-Difluorodeoxycytidine 5′-monophosphate (DFdCMP)	http://www.drugbank.ca/metabolites/DBMET01145
		Difluorodeoxyuridine monophosphate	http://www.drugbank.ca/metabolites/DBMET00694
18	Idarubicin	Idarubicin	42890
		Idarubicinol	13229553
		Idarubicinone (Idarubicin aglycone)	124720
19	Ifosfamide	Ifosfamide	3690
		4-Hydroxy Ifosfamide	308171
		Isophosphamide mustard	100427
		2-Dechloroethylifosfamide	119105
		3-Dechloroethylifosfamide	114861
20	Imatinib	Imatinib	5291
		*N*-Desmethyl Imatinib	9869737
		Imatinib (Pyridine)-N-oxide	9827642
		Imatinib (Piperidine)-N-oxide	29982268
21	Lapatinib	Lapatinib	208908
		Quinoneimine	102284669
22	Methotrexate	Methotrexate	126941
		7-Hydroxymethotrexate	5484402
		2,4-Diamino-N10-methylpteroic acid (DAMPA)	71315111
		7-Hydroxy DAMPA (2,4-Diamino-N10-methylpteroic acid)	29981388
		Methotrexate polyglutamate	4112
23	Mitomycin	Mitomycin	5746
		1,2-*cis*- and *trans*-2,7-Diamino-1-hydroxymitosene	13817091
		2,7-Diaminomitosene	4210
24	Mitoxantrone	Mitoxantrone	4212
		Mitoxantrone monocarboxylic acid	126803
		Mitoxantrone dicarboxylic acid	126805
25	Nilotinib	Nilotinib	644241
		Nilotinib N-oxide	71750948
		Nilotinib glutamate	86688190
26	Paclitaxel	Paclitaxel	36314
		6-alpha-hydroxy Paclitaxel	10056458
		3′-*p*-hydroxy Paclitaxel	3081785
		6-alpha, 3′-*p*-dihydroxy Paclitaxel	http://www.drugbank.ca/metabolites/DBMET00774
27	Pazopanib	Pazopanib	10113978
		Hydroxy Pazopanib	72942038
		*N*-Demethyl Pazopanib	68319455
28	Sorafenib	Sorafenib	216239
		Sorafenib N-oxide	9826472(CID)
		Sorafenib beta-d-glucuronide	http://www.drugbank.ca/metabolites/DBMET01001
		Pyridine N-oxide glucuronide	http://www.drugbank.ca/metabolites/DBMET00994
29	Sunitinib	Sunitinib	5329102
		*N*-Desethyl Sunitinib	10292573
30	Tamoxifen	Tamoxifen	2733526
		*N*-Desmethyl Tamoxifen	3032890
		*N*,*N*-Didesmethyl Tamoxifen	71316031
		(*Z*)-Endoxifen	10090750
		4-Hydroxy Tamoxifen	449459
		Tamoxifen N-oxide	3033895
		*N*-Desmethyl-droloxifene	3035880
31	Temsirolimus	Temsirolimus	6918289
		Sirolimus	5284616
32	Thalidomide	*R*(+) Thalidomide	75792
		*S*(−) Thalidomide	92142
		5-Hydroxythalidomide	5743568
		5′-hydroxy-thalidomide	9878646
		*N*-(*o*-carboxybenzoyl)-glutamic acid imide (glutamine)	134736
		Phthaloylglutamine	98204
		Phthaloylisoglutamine	134283
33	Vemurafenib	Vemurafenib	42611257
34	Vincristine	Vincristine	5978
		Vincristine-N-oxide	71752950
		4-Desacetyl vincristine	13131998

### Target prediction using PharmMapper

In order to predict the targets of our drugs and their metabolites, we used PharmMapper Server (http://59.78.96.61/pharmmapper),^17^ which uses reverse pharmacophore mapping approach for the prediction of potential target for a given small molecule. PharmMapper server functions based on a large database named pharmacophore database (PharmTargetDB) which contains over 7000 receptor-based pharmacophore models. For each drug molecule, SDF file was obtained from PubChem and submitted to PharmMapper server and we obtained top 300 candidates.

### Analysis of drug targets and molecular pathway enrichment analysis

Using the drug-target protein list obtained from PharmMapper, a drug–protein matrix was prepared for all individual metabolites of each drug (ESI list 1†). Common/overlapping target proteins for all drug metabolites were listed under the corresponding drug. Among the top 300 targets, based on ranking we consider the top 100 candidates (potential targets) further for the pathway enrichment analysis as described previously.^19^ These targets were subjected to analysis through Database for Annotation, Visualization and Integrated Discovery (DAVID) (https://david.ncifcrf.gov) as described previously.^20^ In DAVID web server, the Kyoto Encyclopedia of Genes and Genomes (KEGG) database module was used to predict the cellular pathway association in which pathway terms enriched significantly (*p* < 0.05) were considered for further discussion.

### Target validation by VEGF phosphoarray and KEA

In order to validate the PharmMapper based prediction method, we intended to explore the cellular signalling targets of 5-FU on human umbilical vein endothelial cells (HUVEC). The primary endothelial cell culture was administrated with 100 μM concentration of 5-FU for 8 hours. After the treatment schedule, cell lysate was collected and processed for the quantification of phosphorylation level in terms of relative abundance at specific phosphorylation sites of enzymes/substrates involved in vascular endothelial growth factor (VEGF) signalling pathway. For this purpose we used VEGF Pathway Phospho Antibody Array containing 185 antibodies (Fullmoon BioSystem, USA) based on the supplier described protocol. Briefly, cells were scraped with lysis buffer and crushed with lysis beads to prepare cell lysate. Proteins were purified by using column provided in the array kit. Protein quantification was done by NanoDrop 2000 (ThermoFisher Scientific). Desirable amount of protein was biotinylated and incubated with antibody coated slides pre-blocked with milk protein. Finally, Cy3-streptavidin was added for the detection of biotin-conjugated antibody. Both control and treatment (5-FU) array slide were processed simultaneously. Fluorescent scanned images were converted in to list of fluorescence intensity signals of each spots in the array. Normalization of the level of phosphorylation was done with its corresponding non-phosphorylated form of target. Fold difference in phosphorylation level between conditions was calculated and 2-fold cut off criteria was applied to categorize up-regulated and down regulated phosphorylation targets between treatment conditions.

In order to explore novel 5-FU targeted kinases, we intended to infer the list of kinases associated with the list of differentially phosphorylated sites by 5-FU treatments. For this purpose we employed kinase enrichment analysis (KEA) – a web-based tool with an underlying database version 2 (http://www.maayanlab.net/KEA2/).^21^ After analysis, only the significantly enriched kinases were listed that are plausibly considered as targets of 5FU. The list of possible 5FU targets were intersected with PharmMapper predicted list for the evaluation of consistency between the prediction method and experimental validation.

### Target validation by DrugBank database

In this approach, we compared the predicted targets list with the existing knowledge of drug target relationship available in DrugBank (www.drugbank.ca).^22^ To achieve this, the list of PharmMapper based predicted targets were intersected with target list provided in DrugBank (web-enabled database containing comprehensive molecular information about drugs, their mechanisms, their interactions and their targets) for the evaluation of consistency between the current prediction method and previous validations.

## Results and discussion

Post-market identification of the adverse drug reactions (ADR) is an important public health issue. Since cytotoxic agents and targeted therapies that are used to treat cancer result in collateral damage to other tissues including the cardiovascular system, a growing number of clinical trials are now studying the long-term side effects of anticancer therapy, specifically cardiovascular events.^5,23^ However, a clear systems-toxicological level understanding on the mechanism of cardiotoxicity is lacking. In the advent of prediction of possible adverse drug reactions (promiscuity), reliable *in silico* screening of drug candidates and prediction of the functional effects of system perturbations using large-scale network is believed to be advantageous.^24,25^ The above fact prompted us to explore the novel adverse mechanisms (cardiotoxic side effect) of few anti-cancer drugs including its major metabolites based on the proteome scale multiple interaction profile on human targets through reverse pharmacophore and pathway enrichment strategies. After extensive literature survey and manual curation, we prioritized thirty four chemotherapeutic drugs of different groups with cardio-vascular side effects that were reported in different case studies and in original research articles. Further, the available pharmacokinetic information of the 34 cardiotoxic chemotherapeutic drugs was obtained for creating the list of drug metabolites. The extensive list of all the study related drug and their metabolites along with their PubChem ID are illustrated in Table 1.

The top 300 target proteins for each drug and their metabolites were successfully predicted through PharmMapper server and the common protein targets for each drug was listed (ESI list 1†). Unified list of top 300 targets of each parent drug and its metabolites considered as the final drug target list. In order to understand the signalling pathways involved, the drug targets were subjected to KEGG pathway enrichment analysis. A list of signalling pathways enriched significantly (*P* < 0.05) and targeted by all the 34 drugs was prepared (ESI list 2:† signalling pathways). The above list indicates that metabolism of xenobiotics by cytochrome P450 is the top most pathway that have been targeted by all the study drugs. In our current focus, we carefully mined the above data for the relationship of enriched pathways with cardiovascular toxicity. Based on literature survey, we prioritized the CVD associated pathways including VEGF signalling pathway, insulin signalling pathway, focal adhesion, ErbB signalling, peroxisome proliferator-activated receptors (PPAR) signalling, renin–angiotensin system, arginine and proline metabolism.

**Fig. 1:**
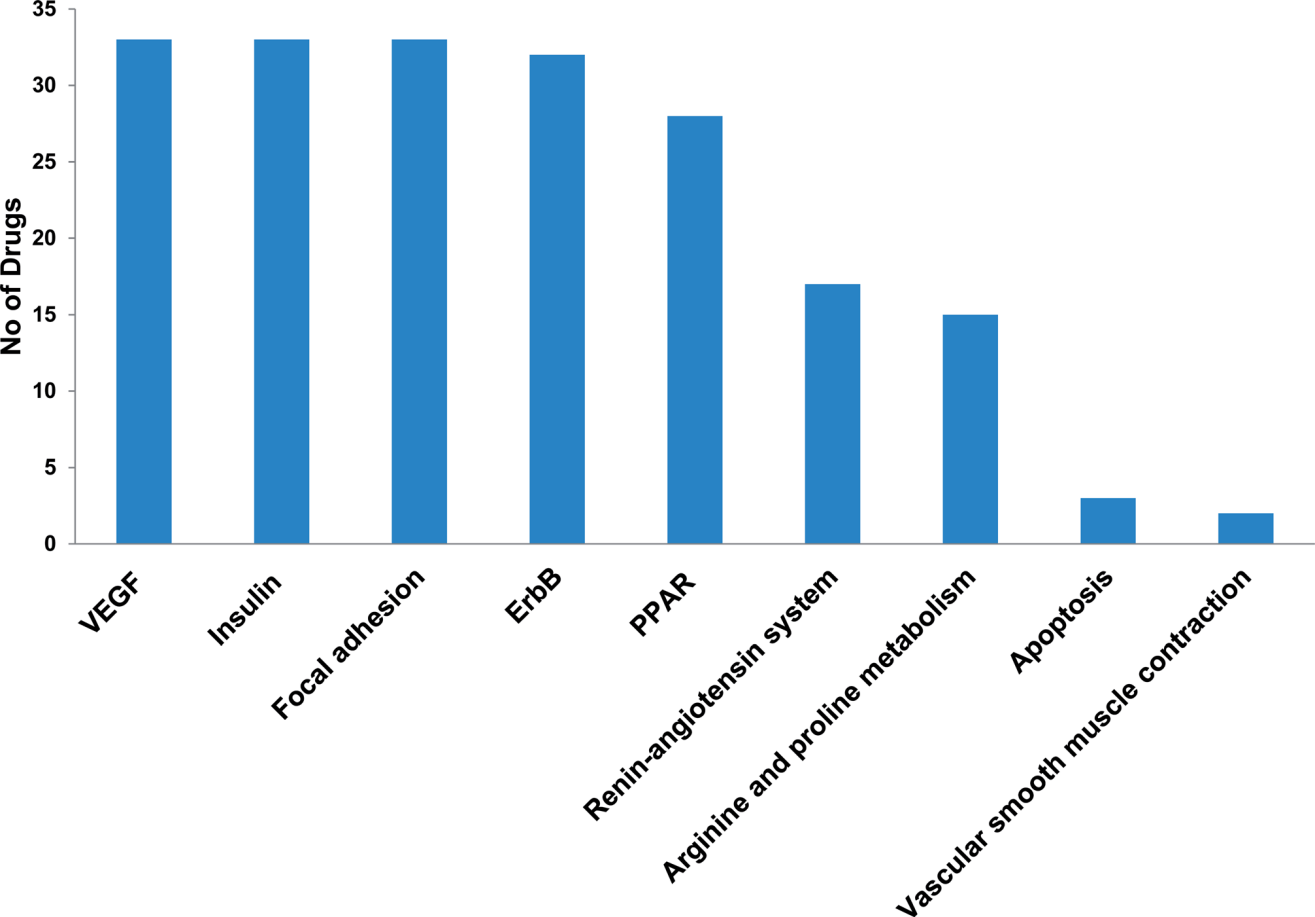
Cardiovascular associated KEGG signalling pathways enriched by cancer drug targets.

**Fig. 2:**
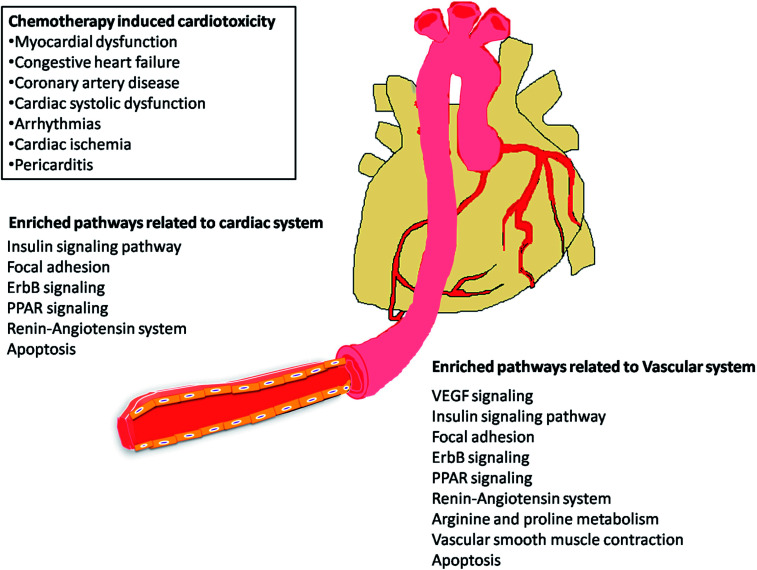
KEGG signalling pathways enriched by cancer drug targets related to cardiac and vascular system.

The most recent ‘European Society of Cardiology’ guidelines indicate that the cardiovascular complications of cancer therapy can be sub classified as (1) cardiac complications including myocardial dysfunction and CHF, coronary artery disease, valvular heart disease, arrhythmias, and pericardial diseases and (2) vascular complications including arterial hypertension, thromboembolic event, peripheral vascular disease and stroke, and pulmonary hypertension.^2,5^ In this connection, current study applies novel methods to predict off-targets and prioritizes the enriched molecular pathways (Fig. 1 and 2) related to the specific cardiovascular events other than intended targets which may add support to the onco-cardiology aspects in terms of prevention, evaluation and monitoring of chemotherapy-induced cardiac toxicity.^26^ Notably, the top pathways enriched by all the 34 drugs were “Metabolism of xenobiotics by cytochrome P450” and “Pathways in cancer”. These two pathways were followed by vascular endothelial growth factor (VEGF) signalling, insulin signalling and focal adhesion which were enriched by all the drugs except cisplatin. VEGF receptors are major players in maintaining the cardiovascular homeostasis^27^ and also endothelial cell functions are highly dependent on VEGF signalling pathway.^28^ Next to VEGF, insulin pathway was also affected by all the drugs except cisplatin in our study. Altered insulin signalling is believed to cause endothelial dysfunction, atherosclerosis^29^ and other cardiovascular pathogenesis including coronary artery disease.^30^ Focal adhesion signalling involving focal adhesion components including β1 integrin, vinculin, focal adhesion kinase (FAK) is indispensible to various cardiac events ranging from embryonic heart development to mechanotransduction.^31^ Further, in cardiac remodeling point of view, FAK knockout mice showed increased cardiac hypertrophy upon Ang II stimulation.^32^ ErbB signalling was also affected by most of the drugs. Previous studies on ErbB signalling components ErbB1, ErbB2, ErbB3 and ErbB4 are differentially expressed and with their primary ligand, neuregulin-1 play substantial roles in embryonic heart development^33^ as well as in adult endothelial and cardiac functions.^34,35^ It is noteworthy that ErbB1/ErbB2 mutant mice suffer from cardiac dysfunction.^36^ The cardio-toxic effects of the well-known breast cancer drug, Trastuzumab was shown to affect cardiac function by interacting with ErbB2.^37^ PPARs signalling play a vital role in cardiovascular physiology and dysfunction including inflammation and circadian rhythm.^38^ PPAR protects cardiac system from oxidative stress, further inhibition of PPAR-α signalling results in cardiac damage.^39^ PPAR signalling was the 5^th^ most common pathway affected by the chemotherapeutic drugs. In the renin–angiotensin (RAS) axis, angiotensin (Ang) II, the main effector of the RAS, is one of the major mediators of vascular remodeling in hypertension and plays critical role in stability of the cardiovascular microenvironment.^40,41^l-Arginine is the main source of nitric oxide (NO) generation *via* NO synthase (NOS) which play vital regulatory role in cardiovascular and renal physiology even under hormonal disorders.^42^ Overall, our results indicate that chemotherapeutic drugs commonly affect specific pathways required for the normal functioning of the cardiovascular system.

**Fig. 3:**
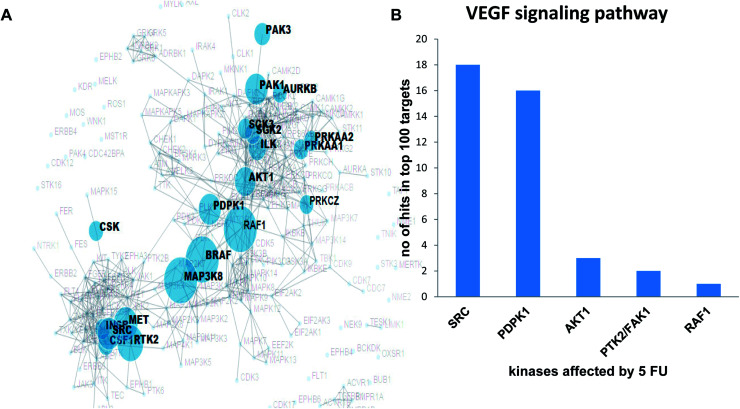
A) Prediction of targets in VEGF signalling pathway through Kinase Enrichment Analysis (KEA) in HUVEC treated with 5 fluorouracil; (B) VEGF signalling pathway associated kinases that are also present in PharmMapper predicted list.

Validation of the overlapping proteins from predicted and experimental targets was achieved by (1) kinase enrichment analysis (KEA) and (2) DrugBank interrogation. In addition, we employed the phosphorylation antibody array to understand the effects of 5-FU on VEGF signalling pathway in terms of modulation of phosphorylation status of specific proteins using HUVEC cells. In order to explore the possible targets of 5-FU (down regulated phosphorylation of substrate due to the inhibition of upstream kinase by 5-FU), we predicted the corresponding upstream kinase through kinase enrichment analysis (KEA). KEA results revealed that upstream kinases such as SRC, AKT1, PDPK1, RAF1, BRAF, MAP3K8, PTK2, MET were significantly associated with a set of under-represented profile of downstream phosphoproteins (which in turn indicate the possibly inhibited upstream kinase targets) due to 5-FU treatment. The enriched kinase nodes are illustrated in Fig. 3A. At the validation level, KEA enriched kinases (list of possibly inhibited kinases with the original *p*-value less than 0.05) were interrogated with the top 100 PharmMapper protein target list of 5-FU which indicated that SRC, PDPK1, AKT1, PTK2/FAK1, RAF1 were present in both the 5-FU target list and PharmMapper predicted list (Fig. 3B). Moreover, through the DrugBank query, the drug targets obtained from PharmMapper were cross-verified with the information provided in DrugBank (targets). Again, the overlapped targets further validate the overall prediction approach (Table 2).

**Table 2:** PharmMapper based predicted targets were intersected with target list provided in DrugBank

S. no.	Drug name	Drug's protein-targets obtained from drug bank database	Our prediction in top 300 (match: ✓ no match: ✘)
1	5-Fluorouracil	Thymidylate synthase	**✓**
2	Bleomycin	DNA ligase 1,	**✘**
		DNA ligase 3	**✘**
3	Busulphan		
4	Camptothecin	DNA topoisomerase 1	**✘**
5	Carboplatin		
6	Cisplatin		
7	Cyclophosphamide		
8	Cytarabine	DNA polymerase beta	**✘**
9	Dasatinib	Tyrosine-protein kinase ABL1,	**✓**
		Proto-oncogene tyrosine-protein kinase Src,	**✓**
		Ephrin type-A receptor 2,	**✓**
		Tyrosine-protein kinase Lck,	**✓**
		Tyrosine-protein kinase Yes,	**✘**
		Mast/stem cell growth factor receptor kit,	**✘**
		Platelet-derived growth factor receptor beta,	**✘**
		Signal transducer and activator of transcription 5B,	**✘**
		Abelson tyrosine-protein kinase 2,	**✘**
		Tyrosine-protein kinase Fyn	**✘**
10	Daunorubicin	DNA topoisomerase 2-alpha,	**✘**
		DNA topoisomerase 2-beta	**✘**
11	Docetaxel	Tubulin beta-1 chain,	
		Apoptosis regulator Bcl-2,	**✓**
		Microtubule-associated protein 2/4/tau,	**✘**
		Nuclear receptor subfamily 1 group I member 2	**✓**
12	Doxorubicin	DNA topoisomerase 2-alpha	**✘**
13	Epirubicin	DNA topoisomerase 2-alpha,	**✘**
		Chromodomain-helicase-DNA-binding protein 1	**✘**
14	Erlotinib	Epidermal growth factor receptor,	**✓**
		Nuclear receptor subfamily 1 group I member 2	**✓**
15	Etoposide	DNA topoisomerase 2-alpha,	**✘**
		DNA topoisomerase 2-beta	**✘**
16	Everolimus	Serine/threonine-protein kinase mTOR	**✘**
17	Gemcitabine	Ribonucleoside-diphosphatereductase large subunit,	**✘**
		Thymidylate synthase,	**✓**
		UMP-CMP kinase	**✘**
18	Idarubicin	DNA topoisomerase 2-alpha	**✓**
19	Ifosfamide		
20	Imatinib	BCR/ABL fusion protein isoform X9,	**✘**
		Mast/stem cell growth factor receptor kit,	**✘**
		RET proto-oncogene,	**✘**
		High affinity nerve growth factor receptor,	**✘**
		Macrophage colony-stimulating factor 1 receptor,	**✘**
		Platelet-derived growth factor receptor alpha/beta,	**✘**
		Epithelial discoidin domain-containing receptor 1,	
		Tyrosine-protein kinase ABL1	**✓**
21	Lapatinib	Epidermal growth factor receptor,	**✓**
		Receptor tyrosine-protein kinase erbB-2	**✘**
22	Methotrexate	Dihydrofolate reductase	**✓**
23	Mitomycin		
24	Mitoxantrone	DNA topoisomerase 2-alpha	**✘**
25	Nilotinib	Tyrosine-protein kinase ABL1,	**✓**
			**✘**
		Mast/stem cell growth factor receptor kit	
26	Paclitaxel	Apoptosis regulator Bcl-2,	**✓**
		Tubulin beta-1 chain,	
		Nuclear receptor subfamily 1 group I member 2,	**✓**
		Microtubule-associated protein 4/2/tau	**✘**
27	Pazopanib	Vascular endothelial growth factor receptor 1/2/3,	**✓**
		Platelet-derived growth factor receptor alpha/beta,	**✘**
		Mast/stem cell growth factor receptor kit,	**✓**
		Fibroblast growth factor receptor 3,	
		Tyrosine-protein kinase ITK/TSK,	**✘**
		Fibroblast growth factor 1,	**✓**
		SH2B adapter protein 3	**✘**
28	Sorafenib	Serine/threonine-protein kinase B-raf,	**✘**
		RAF proto-oncogene serine/threonine-protein kinase,	**✓**
		Vascular endothelial growth factor receptor 3/2/1,	**✓**
		Receptor-type tyrosine-protein kinase FLT3,	**✘**
		Platelet-derived growth factor receptor beta,	**✘**
		Mast/stem cell growth factor receptor kit,	**✘**
		Fibroblast growth factor receptor 1,	**✓**
		Proto-oncogene tyrosine-protein kinase receptor Ret	**✘**
29	Sunitinb	Platelet-derived growth factor receptor beta,	**✘**
		Vascular endothelial growth factor receptor 1/2/3,	**✓**
		Mast/stem cell growth factor receptor kit,	**✘**
		Receptor-type tyrosine-protein kinase FLT3,	**✘**
		Macrophage colony-stimulating factor 1 receptor,	**✘**
		Platelet-derived growth factor receptor alpha	**✘**
30	Tamoxifen	Estrogen receptor alpha,	**✓**
		Estrogen receptor beta,	**✓**
		3-Beta-hydroxysteroid-Delta(8), Delta(7)-isomerase,	**✘**
	
		Protein kinase C	**✘**
31	Temsirolimus	Serine/threonine-protein kinase mTOR	**✘**
32	Thalidomide	Protein cereblon,	**✘**
		Tumor necrosis factor,	**✘**
		Nuclear factor NF-kappa-B p105 subunit,	**✘**
		Fibroblast growth factor receptor 2,	**✘**
		Prostaglandin G/H synthase 2,	**✘**
		Nuclear factor kappa-light-chain-enhancer of activated B cells,	**✘**
		Alpha1-acid glycoprotein	**✘**
33	Vemurafenib	Serine/threonine-protein kinase B-raf	**✓**
34	Vincristine	Tubulin beta chain,	**✘**
		Tubulin alpha-4A chain	**✘**

In the validation aspect, we intend to point out that the PharmMapper protocol have been successfully utilized in more than two hundred articles after the first benchmarking test of Tamoxifen on finding the proper targets (proof of concept) among the top 300 pharmacophore candidates.^17^ Additionally, in the current study, we have support for the PharmMapper target prediction by (1) comparing DrugBank curated protein target list with PharmMapper-top 300 targets (2) comparing 5FU modulated VEGF signalling kinase list obtained from experimentally assisted kinase enrichment analysis with top 100 targets of 5FU from PharmMapper. DrugBank which combines detailed drug data with comprehensive drug target and drug action information drives us to indicate the positive overlapping nature of PharmMapper predicted targets with DrugBank primary/secondary targets for the following drugs including Idarubicin, 5-fluorouracil, Methotrexate, Paclitaxel, Docetaxel, Lapatinib, Sorafenib, Imatinib, Vemurafenib, Nilotinib, Pazopanib, Dasatinib and Tamoxifen. Further, our second method, prediction of targets was through Kinase Enrichment Analysis (KEA), which utilizes the prior knowledge of kinase–substrate interactions and links the lists of mammalian proteins/genes with the kinases that phosphorylate them.^21^ Similarly, a previous study utilized the kinase–substrate enrichment analysis (KSEA) to systematically infer the activation of kinase pathways from mass spectrometry-based phosphoproteomic analysis of Lapatinib resistance cells and acute myeloid leukemia (AML) cells.^43,44^ In this study, we utilized similar template in order to explore the possible kinase targets of 5-FU using VEGF array. We explored the upstream kinases that are believed to phosphorylate the VEGF signalling components that were down phosphorylated after 5-FU treatments in HUVEC. Among the 85 VEGF array phosphorylation substrates, 20 of them were found to be down regulated which in turn drove us to explore the upstream kinase through KEA as RAF1, PTK2/FAK1, SRC, PDPK1, and AKT1. This study was also successful in finding the predicted down regulated kinases as potential targets among the top 100 targets hits from PharmMapper database. The above evidence enrich support to the proof-of-concept of reverse pharmacophore mapping based prediction of targets which in turn strongly supports the proteome-wide prediction of cardio toxic mechanism of anti-cancer drugs before entering into the wet lab experiments. As pointed out in^45^ and demonstrated in a series of recent publications,^46–67^ user-friendly and publicly accessible web-servers represent the future direction for developing practically more useful prediction methods and computational tools. Actually, many practically useful web-servers have increasing impacts on medical science,^16^ driving medicinal chemistry into an unprecedented revolution,^68^ we shall make efforts in our future work to provide a web-server for the prediction approaches presented in this paper.

## Conclusion

In conclusion, the current study provides a detailed analysis on off-target-pathway-cardiovascular homeostasis relationship of anti-cancer drugs related to pathophysiology. Limitations of the current study are (1) the proteome target/background list is not the whole human proteome list which is restricted to the targets available in PharmMapper target list. (2) The study did not explore the relationship of drug-target in terms of stimulatory or inhibitory interaction and only predicted the interaction potential. Because of limited prediction potential we cannot absolutely prioritize the cardiotoxic/non cardiotoxic nature of drugs, whereas this data can be a reference model to partially understand the cardiotoxic mechanism of anti-cancer drugs.

## Conflicts of interest

All authors reviewed critically and refined the manuscript. All authors declare that there is no conflict of interest.

## Funding Sources

SG acknowledges financial support of INSPIRE programme sponsored and managed by the Department of Science and Technology. JM is thankful to Science and Engineering Research Board for young scientist award. LS convey her thanks to Department of Biotechnology for the fellowship. PG acknowledges financial support from UGC. SC is grateful to University Grants Commission (UGC) – Faculty Recharge Programme. All funding agencies are the different wings of Government of India.

## Supplementary Material

RA-008-C8RA02877J-s001
